# The Oral Microbiota in Valvular Heart Disease: Current Knowledge and Future Directions

**DOI:** 10.3390/life13010182

**Published:** 2023-01-08

**Authors:** Ecaterina Neculae, Evelina Maria Gosav, Emilia Valasciuc, Nicoleta Dima, Mariana Floria, Daniela Maria Tanase

**Affiliations:** 1 Department of Gastroenterology, “Grigore T. Popa” University of Medicine and Pharmacy, 700115 Iasi, Romania; 2 Institute of Gastroenterology and Hepatology, “Sf. Spiridon” County Clinical Emergency Hospital Iasi, 700111 Iasi, Romania; 3 Department of Internal Medicine, “Grigore T. Popa” University of Medicine and Pharmacy, 700115 Iasi, Romania; 4 Internal Medicine Clinic, “St. Spiridon” County Clinical Emergency Hospital Iasi, 700111 Iasi, Romania

**Keywords:** oral microbiota, dysbiosis, valvular heart disease, therapeutic strategies

## Abstract

Oral microbiota formation begins from birth, and everything from genetic components to the environment, alongside the host’s behavior (such as diet, smoking, oral hygiene, and even physical activity), contributes to oral microbiota structure. Even though recent studies have focused on the gut microbiota’s role in systemic diseases, the oral microbiome represents the second largest community of microorganisms, making it a new promising therapeutic target. Periodontitis and dental caries are considered the two main consequences of oral bacterial imbalance. Studies have shown that oral dysbiosis effects are not limited locally. Due to technological advancement, research identified oral bacterial species in heart valves. This evidence links oral dysbiosis with the development of valvular heart disease (VHD). This review focuses on describing the mechanism behind prolonged local inflammation and dysbiosis, that can induce bacteriemia by direct or immune-mediated mechanisms and finally VHD. Additionally, we highlight emerging therapies based on controlling oral dysbiosis, periodontal disease, and inflammation with immunological and systemic effects, that exert beneficial effects in VHD management.

## 1. Introduction

The oral cavity is the house of a complex and divergent microbiota community that plays a vital role in an individual’s general homeostasis [1]. The mouth is one of the most colonized parts of the body, possessing after the intestinal system the second largest and most diverse microbiota, including bacteria, viruses, protozoa, and fungi [2]. The oral cavity represents one of the leading portals of entry for several microorganisms. Its microbiota variability depends on specific mouth locations, such as the teeth, periodontal pocket, tongue, cheek, palate, and saliva [3]. Depending on the type of bacterial imbalance with improper homeostatic function, the dysbiosis that occurs can lead to different oral pathologies [4]. Several modifiable factors, including salivary gland impairment, poor oral hygiene, gingival inflammation, dietary habits, and smoking, may lead to oral dysbiosis. This can cause a localized oral inflammatory state, with subsequently chronic low-grade inflammation whose alleviation is not possible in the presence of dysbiosis [5]. Indeed, oral microbiota dysbiosis originates from two primary oral infections, namely caries and periodontitis [6,7]. Along with the immuno-inflammatory axis [8], oral dysbiosis represents a new pathogenic risk factor for the development of cardiovascular diseases (CVDs) [9], such as atherosclerosis, coronary disease, and valvular heart disease (VHD) [10]. Myriad research revealed that if the symbiotic relationship between the resident microbiome breaks down, minor components of the microbiota can outcompete the beneficial bacteria [11]. This process increases the risk of translocating various harmful microbes from the oral cavity to the normal or already affected heart valves, with further permanent damage [12]. An altered heart, for example, secondary to post-surgery (implantation of an artificial heart valve or pacemaker), has a modified tissue structure with increased roughness that promotes easy bacterial attachment to cardiac valves or the heart lining [12]. Therefore, this review aims to describe the pathophysiological link between dysbiotic oral microbiota and VHD, focusing on the microorganisms involved, prevention, and the potential therapeutic applications of oral dysbiosis in VHD management.

## 2. Links between Dysbiotic Oral Microbiota and Valvular Heart Disease Development

### 2.1. Oral Dysbiosis

The human microbiota represents 90% of the cells in the human body. This caused scientific communities worldwide to realize that variations in the composition and structure of the major parts of the human microbiota (e.g., gut, skin, oral) play a major role in the development of different pathologies [13]. The term oral microbiome defines microorganisms that inhabit the human oral cavity. Immediately after birth, the acquirement of the microorganisms that compose the microbiota begins [14]. Oral microbiota structure varies due to the host’s genetics, and external factors (environment, diet, oral hygiene, physical activity, smoking, medication use, and other exogenous microorganisms) [15]. Any alterations of the oralome and of microbiome matrix composition are defined as “dysbiosis”. The “oralome” is defined as the vast dynamic interactions coordinated between the ecological community of the oral microbiome and the host. The symbiotic relationship with the host is driven by the oral composition of this complex system. Oral dysbiosis is a significant injurious switch in the relative abundances of beneficial and harmful microbes in the mouth [16].

As already mentioned, oral dysbiosis is a known risk factor for metabolic and CVDs and, unfortunately, more than 50% of oral microorganisms cannot be cultivated or identified [17]. This is why different culture-independent methods for detection have been developed in the past years to better understand the interaction of synergistic and antagonistic interspecies alongside the imbalance of different bacteria that contribute to disease occurrence [18]. Out of the 700 bacterial species discovered in the oral cavity, more than 300 have been nurtured [19,20]. Using microbiological methods, immunohistological structural analysis, and next-generation sequencing detection of the bacterial metagenome, researchers noted that the pathogenic spectrum of VHD includes mainly Gram-positive and Gram-negative bacteria [21]. Hence, the crossing of metabolomics and microbiota research and new detection methods may bring additional information crucial to discover novel therapies that can reduce disease susceptibility [22].

#### 2.1.1. The Role of Salivary Microbiota in Oral Dysbiosis

Salivary microbiota is considered to mirror the inhabiting oral mucosa and the teeth. The organic biofilm that protects teeth from acidic and mechanical aggressions promotes the addition of heterogeneous aerobic and anaerobic bacteria. The saliva plays a significant role in modeling the structure of the microbiome through immune components with antibacterial properties, such as lysozyme, lactoferrin, immunoglobulins, histidine-rich proteins, and the peroxidase system [23]. Furthermore, glycoproteins found in the saliva composition ensure the bacteria’s nutrition, and proteins, such as mucin, that are found in saliva can stop the adherence of microorganisms to oral surfaces through aggregating and binding mechanisms. All these elements contribute to maintaining a balance in the biofilm and microbiome structures. Even small changes or insufficient salivary production can lead to oral dysbiosis [23,24]. 

The most common phyla identified at the salivary level are *Actinobacteria, Bacteroides, Firmicutes, Fusobacteria, Proteobacteria, Spirochaetes*, and *Saccharibacteria* (formerly known as TM7). Globally, members of the population share similar oral microorganisms [23]. This indicates there is no apparent geographical distribution [25]. Although individual differences are considered normal [26], salivary microbiota dysbiosis can be used in identifying different diseases, including endothelial dysfunction [27], hypertension, or pulmonary disorders, such as sleep apnea [28]. 

#### 2.1.2. The Role of Dental Plaque in Oral Dysbiosis

Periodontal disease and dental caries and are the most prevalent microbially mediated oral affections that burden humans. Dental plaque is currently described as a polymicrobial biofilm, characterized by as a colony of microbial cells integrated in an extracellular matrix. The structure of dental plaque biofilms is steered by various synergistic inter-taxon interactions, ecological succession, and dynamic environmental, physical, and chemical interactions [29,30]. Dental plaque is classified into two main categories, namely below the gingival margin (subgingival) and above the gingival margin (supragingival). The former contains a variety of bacteria, including *Actinobacteria* and *Firmicutes* [30]. The *Streptococcus* genus, represented by *S. gordonii*, *S. mitis*, *S. mutans* and *S. sanguis*, remains the main bacteria identified from dental plaque [31]. Other bacteria isolated often from the dental plaque are *Actinomyces*, *Campylobacter*, *Capnocytophaga*, *Fusobacterium*, *Neisseria*, and *Veillonella* [32,33]. 

Imbalance in the supragingival dental plaque composition is one of the first steps in developing disease conditions [26]. A shift in supragingival community composition from one that is beneficial to one that mediates diseases endorses the formation of caries. Caries result from a distribution of the supragingival biofilm frequently associated with high sugar intake or neglecting oral hygiene. This leads to the multiplying of Gram-positive bacteria, such as *Actinomyces*, *Lactobacillus*, and *Streptococcus mutans*. In addition, beneficial bacteria proliferation and activity are reduced due to the acidification of the oral cavity medium [34,35,36]. *Streptococcus mutans* especially ferments sucrose to produce ATP and lactic acid that accumulate in time and creates an acidic local environment that promotes caries. Bacteria binds to the saliva-coated tooth, grow rapidly, and antagonize competitors with the local secretion of hydrogen peroxide, which further leads to highly localized acidification and tissue demineralization [37].

Through computer technology and other novel approaches, researchers may discover novel antimicrobial agents targeting the biofilms of most relevant bacteria involved in oral dysbiosis [38].

#### 2.1.3. The Role of Oral Mucosa Colonization in Oral Dysbiosis

The oral mucosa colonization is limited, being mostly related to halitosis without influencing systemic pathologies. That being said, it is known to influence the development of the two most common oral disorders, namely dental caries, as was previously discussed, and periodontal diseases [14]. 

#### 2.1.4. The Role of Periodontitis in Oral Dysbiosis

Chronic periodontitis is the most frequent oral infection associated with CVDs, particularly endodontic infection [39]. The presence in the subgingival biofilm of Gram-negative anaerobic bacteria (*Fusobacterium nucleatum*, *Aggregatibacter actinomycetemcommitans*, *Prevotella intermedia*, *Tannerella forsythia, Porphyromonas gingivalis*, and *Treponema denticola*) has been known to initiate or sustain the progression of the chronic inflammatory process that leads to periodontitis [40]. Recent studies have shown that periodontal dysbiosis results from changes in the number and function of the commensal bacteria. There is little influence from new species that colonize the oral cavity [5,41,42,43]. This not only contributes to the development of a variety of systemic pathologies, but also to their aggravation [44].

According to the consensus report by the European Federation of Periodontology (EFP) and the American Academy of Periodontology, periodontitis is also a chronic non-communicable disease (NCD) [10]. Severity and progression rate of periodontitis is an independent risk factor for CVD, such as hypertension, atherosclerosis [45], or VHD [46]. Additionally, Sia SK et al. [46] shows that intensive treatment of periodontitis significantly lowers the risk for VHD development. 

### 2.2. Detection of Oral Bacterial Species in Cardiovascular Specimens

#### 2.2.1. Detection of Oral Bacterial Species in Patients with Rheumatic Heart Disease (RHD)

It is known that CVDs, including heart valve dysfunctions, represent the leading mortality cause worldwide. Approximately 17.9 million people died due to CVDs (32% of deaths globally) in 2019, according to the WHO (World Health Organization) [47]. Although the main causes of CVDs are metabolic or unhealthy lifestyles, such as a high-fat diet, physical inactivity, obesity, alcohol use, and smoking, in the past years, much of the focus has been on the role of gut microbiota in different pathogenic disorders. In a very recent study, the authors analyzed the influence of not only gut but also oral microbiota in patients with rheumatic heart disease (RHD), classically known to be the result of group A *Streptococcus* infection [11]. The richness in beta diversity of the salivary microbiota was higher than the control group, and the bacteria identified in the subgingival plaques (*Corynebacterium*, *Lachnoanaerobaculum* and *Roseburia*) were correlated with RHD severity. Interestingly, *Streptococcus* genus was increased in both saliva and subgingival plaques. Microbiota identified at the mitral valve level partially overlaps with the one in the oral cavity. This bacterial translocation creates antigens that induce an autoimmune response against the cardiac tissue, contributing to the evolution of RHD. Patients who presented alterations in one or both microbiotas were more likely to experience a more severe RHD due to translocation to the mitral valves [11]. Oral bacteria could enter the bloodstream during invasive dental procedures and while performing daily tasks, such as eating or brushing the teeth [48]. Although the bacteria found in the mitral valves were partially common with that from the oral microbiota, *Campylobacter* was the one that mainly represented the oral to valve translocation [11]. Furthermore, chronic periodontitis patients have a local oxidative stress profile which is enhanced by the presence of RHD [48]. 

#### 2.2.2. Detection of Oral Bacterial Species in Patients with Valvular Disease

Raffaelli et al. [49] focused on identifying the DNA from periodontal bacteria in aortic valves concomitant to whole-body samples in patients that underwent aortic valve replacement interventions. There was no correlation between the oral pathogens and the valve specimens or blood samples. Perhaps due to raised local aortic valve blood pressure, adhesion and proliferation of the bacteria were not possible. Therefore, an indirect factor caused by inflammation markers generated by bacteria was most likely one of the leading causes of aortic stenosis. Nakano et al. [50] tried to identify the role of causative pathogens in CVDs of cariogenic oral bacteria (*Streptococcus mutans*) by heart valve specimens and atheromatous plaque detection. They completed their research by extracting bacterial DNA from dental plaque and cardiovascular tissues (aortic and mitral valve specimens, aortic aneurysmal wall specimens). *Streptococcus mutans*, followed by *Aggregatibacter actinomycetemcomitans*, were the main oral bacterial species involved in developing CVDs [51].

The most common valvular disease is aortic stenosis, usually due to calcification. Cohen et al. [52] observed in animal models how oral bacteria contributes to recurrent low-grade endocarditis that causes aortic valves calcifications. They found that bacteria, such as *Corynebacterium matruchotti*, *Streptococcus mitis*, and *Streptococcus sanguis* II, can initiate calcification by mineralizing. The group inoculated with *Corynebacterium matruchotti* and *Streptococcus sanguis* II colonies had the most affected aortic valves by calcifications, followed by the group inoculated with *S. sanguis* II. The control group and the one injected with *C. matruchotti* had no traces of aortic valve calcification [52].

#### 2.2.3. Detection of Oral Bacterial Species in Patients Undergone Cardiac Surgical Procedures

A recent study via utilization of 16S rRNA gene amplification detected the presence of certain bacteria in heart valve samples from patients that underwent surgery for heart valve replacement. Most of them were diagnosed with aortic stenosis, followed by mixed aortic valve disease (a combination between aortic regurgitation and aortic stenosis), aortic valve insufficiency, and bicuspid aortic valve. Of the seven Gram-positive bacteria species identified, only *Streptococcus sanguinis*, *Streptococcus oralis*, and *Streptococcus* sp. were typical oral bacteria. *Cutibacterium acnes* was the most detected bacterial species [53]. In a study published by Moreno et al. *Cutibacterium acnes* was identified in 12% of the replaced heart valves. However, this bacterium is regarded as normal skin flora and a commensal of the oral cavity [54]. 

Oliveira and colleagues conducted a more conclusive study where they analyzed samples from patients with different heart valve diseases, consisting of supragingival and subgingival plaque, saliva, and cardiac valve tissue. Mitral regurgitation was the main cause that led to valve replacement surgery in 11 heart valves collected from the patients, followed by aortic stenosis (10 heart valves), aortic insufficiency (8 heart valves), mitral stenosis (8 heart valves), double aortic lesion (6 heart valves),and double mitral lesion (4 heart valves). The presence of oral bacteria was found in 42 of the 47 heart valve samples. *Streptococcus mutans* was found in 89.3% of the valves, followed in a much lower percentage by *P. intermedia*, *T. denticola*, and *P. gigngivalis*. The detection of *S. mutans* in dental plaque and saliva samples was lower than in cardiac valve samples. This was mainly because some of the patients included in the study had dental caries. Furthermore, *S. mutans* survives in the bloodstream before attaching to extracellular matrix components [55]. It has been reported in various studies that *S. mutans* obtains advantages while passing through the bloodstream. Plasma components influence the expression of specific *S. mutans* genes [56]. Here, AtlA is a fibronectin-binding protein that confers to *S. mutans* the ability to avoid phagocytosis [57]. After it reaches the heart valve tissue, it invades the endothelial cells with subsequent inflammation, producing cytokines and platelet aggregation [58]. 

In another study conducted on patients undergoing aortic valve replacement, Pardo et al. [59] observed the presence of the same oral pathogens in both the aortic valvular samples and the oral cavities of the patients. Seven patients were edentulous, fifteen presented severe to moderate periodontitis, and only four were considered orally healthy. Oral pathogens were detected in seven out of nine valve specimens from patients with periodontal disease. The final results were consistent with the previous findings and studies. This strengthens the possibility that bacteria found in plaque deposits enter the blood circulation, with *Porphyromonas gingivalis* and *Prevotella pasteri* being statically significant. *Streptococcus mutans* was the most frequent non-periodontal microorganism identified at the valvular level, a species commonly found in other studies [59].

Oral dysbiosis leading to local and systemic diseases is causing the oral microbiome to become a target for a new field of research. Despite their promising role, the results obtained from different studies are not consistent, by reason of the different detection techniques used, or the standardization methods and sample size [60]. Therefore, to further identify the mechanism behind oral dysbiosis and VHD association, and to identify targeted therapies and personalized medicine, larger sample size-studies are required.

### 2.3. Mechanisms That Link Oral Dysbiosis to Valvular Heart Disease

The mechanisms behind the complex interrelationship between oral pathogen-induced inflammation and CVD onset and/or progression have yet to be understood completely [44]. As seen, a gamut of evidence linked oral dysbiosis to various CVDs, including heart valve disease and aortic aneurysms [61]. Supposedly, invasive procedures, such as subgingival periodontal instrumentation/extractions, and even simple everyday tasks, such as chewing or flossing, can induce the dissemination of oral bacterial components or toxins into the bloodstream. This mechanism has, as a result, transient or even prolonged bacteremia generated by direct injury at the oral level [62].

Another factor is the immune system’s reaction to chronic periodontitis or endodontic infections. There is a greater risk of bacteremia episodes in those with periodontal disease. This causes the bloodstream elevation of inflammatory mediators, such as C-reactive protein and lipopolysaccharides (LPSs) levels [63]. The inflammation markers produce a response that can cause heart tissue damage by an indirect systemic effect [64]. These LPSs contribute to the production of proinflammatory cytokines. They are produced mainly by Gram-negative species, such as *Tannerella forsythia, Porphyromonas gingivalis*, and *Treponema denticola* [65]. A cascade reaction is triggered, and prostaglandin E2 and matrix metalloproteinases are produced. This leads to local inflammation, allowing pathogens to enter the systemic circulation [49]. Recent studies focused on identifying periodontal pathogens in the cardiovascular system, including in heart valves, myocardial tissues, and atherosclerotic plaque [66,67]. Apart from identifying deoxyribonucleic acid (DNA) from oral bacteria in atrial and ventricular myocardium, some authors described the detection of LPS-binding protein at this level, giving a better understanding of the histological, immunohistochemical, and biochemical links between periodontal inflammation, cardiovascular conditions, and immune responses [68].

**Figure 1:**
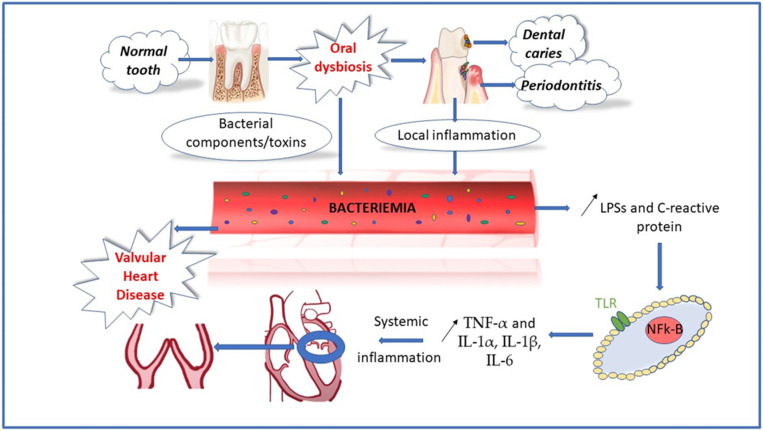
Association between oral dysbiosis and valvular heart disease.

The main path of activation of the immune defense mechanisms is considered through pathogen-associated molecular patterns (PAMPs). These are represented by surface molecules of Gram-negative species, such as LPSs, that have been mentioned before, or teichoic acid, which is Gram-positive specific. They are recognized through pattern recognition receptors, including Toll-like receptors (TLRs), while the transcription nuclear factor kappa B (NFk-B) generates the synthesis of proinflammatory mediators. This pathway activates the myeloid differentiation protein 88 as an outcome of PAMPs and TRLs interaction. Mitogen-activated kinases, inhibitor of kappa B kinases and Janus kinases, and signal transducers and activators of transcription are pathways through which the inflammatory signal is transmitted to the nucleus of various cells. The protein kinases catalyze the phosphorylation chain reaction which maintains the production of tumor necrosis factor-alfa (TNF-α) and proinflammatory interleukins (IL-1α, IL-1β, IL-6), resulting in low-grade inflammation that leads to systemic diseases [1,69]. A simplified version of all these mechanisms is represented in Figure 1.

## 3. Oral Microbiota Modulation in Valvular Heart Disease–Current and Novel Therapeutic Strategies

Along with the development of new therapeutic perspectives, clinicians should also maintain their attention on the use of existing oral therapies, such as oral and dental health promotion, in controlling oral dysbiosis that can enhance the beneficial effect of already known treatments [70]. While cardiovascular disease is the leading cause of death worldwide, oral pathologies are some of the most common diseases and are closely linked to daily activities. That being said, the oblivion of oralome is not a viable option, since non-pathogenic bacteria can bring beneficial effects to the host [71]. 

As it is proven that deficient oral health can lead to systemic diseases, in the past years, researchers exerted additional efforts in finding oral biomarkers that could indicate and improve the prevention of systemic illnesses, and novel therapeutic targets for disease management. Nonetheless, the link between periodontal inflammation and systemic/local CVDs is still not fully understood [72]. Strategies that involve pharmacological treatment with anti-inflammatory agents which can prevent or reverse calcific aortic valve disease (CAVD) are currently being investigated; however, valve replacement remains the only treatment option for patients with severe VHD [73]. 

Even if there are no specific agents that could target oral dysbiosis and VHD, it is worth discussing traditional oral hygiene methods with an impact on oral dysbiosis, novel therapeutic strategies, and modifiers of the inflammatory response, which hold future potential in VHD treatment.

### 3.1. Current Therapeutic Strategies

#### 3.1.1. Host Modulation Therapy

Host modulation therapy is a new developing concept in which drug treatments are used as an additional therapeutic option to conventional periodontal treatments. It aims to reduce the destructive side of the host’s inflammatory response. The oral microbiome mediates microbial interspecies interactions and their impact on the health of the oral cavity. Thereby, it creates an interdependent relationship with the host [74]. There are two main approaches in host modulation therapy. One inhibits the inflammatory response, and the other modulates the collagenolytic response, not only in the soft tissue but also in the alveolar bone [75]. Maintaining the balance between pro-inflammatory and anti-inflammatory mediators is the primary target of host modulation therapy. This leads to inflammation resolution that restricts disease development and even induces periodontal tissue repair. There has been a continuous search for alternative treatments, considering the severe side effects of nonsteroidal anti-inflammatory drugs [76]. New compounds called resolvins, including docosahexaenoic and eicosapentaenoic acids, as well as derivatives of omega-3 fatty acids and lipoxins resulting from arachidonic acid, have been considered [75]. Administering a subantimicrobial dose of doxycycline inhibits the matrix metalloproteinases and, therefore, is used as a host response modulator in periodontitis [77]. There are also chemically modified tetracyclines. One, in particular, tetracycline-3, has been tested on patients with periodontal disease but has not been approved yet [75]. Anticollagenolytic compounds are mostly chemical derivates of curcumin. The most representative is a tiketonic phenylaminocarbonyl curcumin that inhibits the matrix metalloproteinase known as curcumin-2.24 [74]. Host modulators have been tested only in vitro and through animal model studies. Seven compounds, called sirtuins, particularly the nuclear ones (1, 2, 6, and 7), have been studied regarding inflammatory diseases, including periodontal ones [76,77]. An essential component of red wine, resveratrol, activates sirtuin 1. In a study conducted by Ikeda et al. [78] on a murine model with induced periodontitis, resveratrol showed its therapeutic potential by inhibiting periodontal breakdown. In 2020, Batool et al. [79] published an article describing an oxygen carrier (M101). It was isolated from *Arenicola mariana*, and its effects were studied in vitro on *Porphyromonas gingivalis*-infected epithelial cells. There was a reduced expression of pro-inflammatory markers. The most relevant ones were TNF-α, IL-1 beta, and receptor activator of nuclear factor kappa beta ligand (RANKL). In addition, the proteome inhibited pro-inflammatory cytokines and chemokine ligands and stimulated pro-healing mediators and immune modulators. It also decreased *P. gingivalis* expression, making it a possible therapeutic agent in *P. gingivalis* infections.

Therefore, host modulation therapy focuses on manipulating the immune response in order to prevent or ameliorate inflammation and tissue damage. It is an adjunct to traditional local therapy in the clinical management of periodontal disease. The latest studies presented a series of emerging compounds, such as resolvins, chemically modified tetracyclines, curcumin derivates, sirtuins, and an oxygen carrier (M101), that show great potential in future treatment use.

#### 3.1.2. Oral Hygiene

Considering the evidence exposed so far in our discussion about the connection of oral dysbiosis to valvular heart disease, it is normal not to exclude the benefits of oral hygiene as a preventive of local/systemic disease method.

Accordingly, daily personal oral hygiene may be considered a key element in preventing periodontal disease, and subsequently diseases with inflammatory substrates [80]. Each medical clinician should advise their patient to consult a dental doctor for professional advice. It is general knowledge that oral health maintenance is based on regular oral hygiene measures, i.e., flossing and brushing teeth, topical use of fluoride, routine dental care, and low cariogenic nutrition [81,82]. Effective brushing, dental floss, or inter-dental brush utilization, and oral rinses will improve oral dysbiosis. Fluorides exert protective and remineralizing effects against caries and dental erosion [83,84]. Furthermore, chlorhexidine with antiseptic and disinfectant actions has shown anti-plaque and antimicrobial oral effects [85,86]. Due to the importance of preventing tooth decay and periodontal disease, daily brushing quality is essential [87].

#### 3.1.3. Nutrition

Microorganisms found at the oral level can easily follow the digestive tract downstream through saliva, linking them to CVDs [88,89]. Diet influences not only oral microbiota composition but also the host’s health. In their article, Zaura et al. revealed significant differences between hunters, farmers, vegetarians, and western dieters regarding oral microbiota [90]. The predominance of *Haemophilus* and *Neisseria* differs between hunters and westerners, while farmers fall in the middle. A high-meat diet benefits oral pathogens; therefore, hunters carry a higher risk of developing oral diseases. Vegetarians also presented an altered microbiota. This included the oral pathogens mentioned before and respiratory tract bacteria, especially *Campylobacter* and *Porphyromonas*. The latest technology has shown that diet patterns play a significant role in maintaining the balance of core species [91]. For example, a comparison between areca and betel nut chewers showed a reduced bacterial diversity in the first group. Meanwhile, the second one revealed the proliferation of *Actinomyces* and *Streptococcus* [92]. 

On the other hand, the inflammatory response caused by periodontal pathogens can be inhibited by alcohol polyphenols. They can be found in apples, cherries, grapes, and red wine. The antibacterial effect of polyphenols is exerted mainly on *S. mutans.* Red wine consumption and the therapeutic use of oenological extracts have demonstrated a potential action against periodontal pathogens, such as *A. actynomycetemcomitans*, *F. nucleatum*, and *P. gingivalis*. This effect was not correlated with the presence of ethanol [93,94]. Therefore, polyphenols are good candidates for natural therapy against oral pathogens. 

Green tea is gaining ground as a potential adjunct in preventing and treating oral and systemic diseases. Catechins are polyphenol components of green tea that contribute to antioxidant activity, stimulating anti-inflammatory action. The most important ones are epigallocatechin-3 gallate and epicatechin-3-gallate. The polyphenol concentration in green tea is 30–40%. It is much higher than that of black tea (only 3–10%). In addition, catechins have impressive antibacterial and antiviral action. It is also essential to mention their antimutagenic and anti-aging properties [95,96].

### 3.2. Novel Therapeutic Strategies

Several “classic” therapies are often insufficient in maintaining the eubiotic state of the oral ecosystem. Thus, new preventive strategies are emerging. In addition, improving and maintaining high levels of oral health should be considered in order to reduce the risk of VHD.

#### 3.2.1. Antibiotic Prophylaxis

Antibiotics have been used in the last century to combat bacterial infections. Continuous research has been conducted to better adapt antibiotic treatments to the patient’s needs [97]. Compared with systemic antibiotics, using drugs targeting specific microorganisms, and, thus, influencing the microbiota, is a newly emerging therapy. There are not enough studies related to the direct effects of antibiotics on the oral microbiota. All guideline committees worldwide recommend antibiotic prophylaxis (AP) for high-risk individuals undergoing invasive dental procedures [98].

Regarding potential risks, conditions for which prophylaxis is still recommended include prosthetic heart valves and RHD in patients at high risk of endocarditis. Furthermore, dentists indicated a high level of concern about the overuse of antibiotics that can cause resistant bacteria and an increased risk of adverse drug reactions [14,99]. This is why the indications of AP were limited, alongside the adaptation of the antibiotic regimens to the most efficient ones [99]. In 2021, the American Heart Association (AHA) issued an update to its 2007 guideline regarding the prevention of Viridans Streptococcal Group Infective Endocarditis (VSG IE) [100]. The 2021 list of cardiac pathologies for which AP is recommended is substantially similar, with some minor additions. The first is that clindamycin is no longer recommended as a pre-operative antibiotic for VGS IE prophylaxis. Recent studies have shown that it often causes frequent severe adverse reactions compared to other antibiotics [101]. Additionally, doxycycline is used as an alternative for patients allergic to penicillin. The AP is recommended for those with cardiac conditions (prosthetic heart valve, history of IE, certain types of congenital heart disease, and cardiac transplantation with heart valve abnormalities). These patients have a higher risk of complications from IE [101,102].

There is still a need for randomized control studies to provide better data on the short and long-term effects of AP administration. In addition, considering the growing number of bacteria with high resistance due to aggressive antibiotic treatments used in the past years, alternative therapies have become increasingly necessary.

#### 3.2.2. Prebiotics and Probiotics

The gut microbiota has been the focus of many studies in the last few years. Given that oral microbiota shares the same principles as the gastrointestinal one, it has been of interest how selected beneficial bacteria can influence inflammation at the periodontal level leading to a better understanding of the prevention of systemic diseases (Table 1 and Table 2) [103]. Probiotics are represented by different microorganisms selected and cultivated to be used as therapeutic agents. Their action is limited due to the host’s genetics, which influences the human body’s multiplication and colonization with beneficial bacteria [104]. They must also meet essential conditions, such as antibacterial action against harmful oral microorganisms, while excluding their own pathogenic gene sequences. In addition, probiotics used in modulating oral microbiota need the ability to penetrate and colonize the epithelial cells while mediating an immune response due to periodontal pathogens [105].

Dental caries and periodontal diseases result from an imbalance in the oral microbiota that contributes to the pathogenicity alongside already-known factors, such as high carbohydrate intake, reduced saliva flow, or plaque accumulation [106]. Prebiotics are food ingredients that are not influenced by digestion. They improve the host’s health by targeting beneficial bacteria and improving their action or contributing to its multiplication [107]. Some studies showed the effect of N-acetyl-D-mannosamine on improving the composition of oral microbiota by sustaining the growth of beneficial bacteria and inhibiting pathogens [108,109]. 

In a study by Esteban-Fernández and colleagues, they found that *Streptococcus dentisani* inhibited the growth of periodontal pathogens, such as *Fusobacterium nucleatum* and *Porphyromonas gingivalis*. It also contributed to the increase in IL-10 cytokine, with anti-inflammatory results, alongside inhibiting interferon-γ expression [110]. *Lactobacillus acidophilus* and *Lactobacillus paracasei* had a beneficial impact on patients with oral inflammation represented by gingivitis and periodontitis by inhibiting the growth of *Staphylococcus aureus* [111,112]. *Lactobacillus* strains, including *L. delbrueckii*, which is found in some yogurt products, inhibit the growth of *P. gingivalis* through hydrogen peroxide production [113]. *Lactobacilli* and *Bifidobacterium* strains modulate the immune and inflammatory host response to gingival epithelial cells infection with *Prophyromonas gingivalis*. A decrease in cytokine levels (IL-1 beta, IL-6) and TNF-α after administrating *Lactobacillus* strains has been noted. Furthermore, they can increase the expression of IL-8 through the activation of the C-X-C motif chemokine ligand 8 gene. This has an anti-inflammatory effect because it cancels out IL-8 degradation caused by *P. gingivalis* [114].

The impact of probiotics has been studied in the peri-implant prevention of infection and inflammation. *Lactobacillus salivarus* has proven its efficiency against *Prevotella intermedia*, *Prophyromonas gingivalis*, *Staphylococcus aureus*, and *Streptococcus salivarius*. *Streptococci* also inhibit the first two by producing organic acids, especially lactate [105]. *Aggregatibacter actinomycetemcomitans* was resistant at all probiotic concentrations used in the study [115]. Fortunately, it can be inhibited by *Lactobacillus bulgaricus* [116]. *Lactobacillus salivarus* can reduce periodontal pathogens from dental plaque, including a decrease in *P. gingivalis* concentration [117]. Furthermore, *P. gingivalis* and *F. nucleatum* are both inhibited by *Lactobacillus rhamnosus* strains [105]. *Lactobacillus fermentum* is used as an aerobe bacteria inhibitor, whereas *Lactobacillus gasseri* targets anaerobes [118]. A strain of *Lactobacillus*, *L. johnsonii*, increases macrophage activation, resulting in the phagocytosis of *A. actinomycetemcomitans* [119]. The effect of oral probiotics that contain a *Streptococcus salivarus* K12 strain has been observed in a recent study published by Babina and collaborators. Oral biofilm was influenced by administrating the probiotic for up to 4 weeks, leading to a decrease in plaque accumulation. Immunoglobulin A (IgA) can be found at the salivary level and has an essential role in caries prevention. Unfortunately, the *Streptococcus salivarus* K12 strain did not impact the salivation rate or the secretion of IgA [120].

A study published in 2020 by Rosier et al. [106] focuses on a new perspective, namely oral bacteria that reduce salivary nitrate. This prevents oral diseases and improves general health by increasing systemic nitric oxide levels. It has been shown that the highest intake of nitrates is from fruits and vegetables, which also prevents the accumulation of carcinogenic nitrite compounds through antioxidants and polyphenols [121]. The human body cells cannot reduce nitrate on their own, but do so through different enzymatic or non-enzymatic processes that produce nitric oxide. Several bacteria, such as *Actinomyces, Corynebacterium*, *Haemophilus*, *Kingella*, *Neisseria*, *Rothia*, and *Veillonella*, are representative for nitrate reduction to nitrite [122]. Nitrate-enhanced supplements have been used as prebiotics to help stimulate the whole nitrate reduction process by the oral microbiota. For example, beetroot consumption increased *Rothia* and *Neisseria* salivary levels [123]. Furthermore, a study by Jockel-Schneider observed that lettuce juice reduces gingival inflammation due to its high nitrate composition [124]. It is considered that nitrate reduction contributes to maintaining oral eubiosis by preventing acidification that could lead to the multiplication of cariogenic bacteria, stimulating oral and general host health. Rosier’s study [106] aimed to identify and isolate oral bacteria with nitrate-reducing proprieties. They have shown that certain strains of *Rothia* enhance the nitrate-reduction capacity of oral communities. *Rothia* isolates directly affect lactate consumption, contributing to pH elevation. Furthermore, prebiotic treatment can be administered as a nitrate or a symbiotic combination with an isolate that reduces nitrate and prevents acidification and lactate accumulation. There is also evidence that nitrate can be converted to nitric oxide or ammonia [125]. Arginine is a known prebiotic that stimulates ammonia production, leading to the alkalinization of the oral cavity environment and the prevention of dental caries [125]. *Lactobacillus brevis* releases arginine deiminase that inhibits the production of nitric oxide. This inhibits vascular permeability, leading to inflammatory cell infiltration [126]. A direct implication between probiotic and prebiotic use in oral dysbiosis leading to a beneficial impact in valvular heart disease is still hard to acknowledge and needs further investigation.

**Table 1:** Prebiotics used in oral dysbiosis treatment.

Prebiotics	Main Action	Other Effects	Year and Reference
N-acetyl-D-mannosamine	Sustains the growth of beneficial bacteria	Inhibits pathogens	2018, [108] 2017, [109]
Beetroot	Increased *Rothia* and *Neisseria* salivary levels	Regulates blood pressure	2018, [123]
Lettuce juice	Reduces gingival inflammation	-	2016, [124]
Arginine	Stimulates ammonia production	Alkalinization of the oral cavity environment and dental caries prevention	2020, [125]

**Table 2:** Probiotics used in oral dysbiosis treatment.

Probiotics	Main Action	Other Effects	Year and Reference
Streptococcus dentisani	Inhibits the growth of *Fusobacterium nucleatum* and *Porphyromonas gingivalis*	Increases IL-10 cytokine production; inhibits interferon-γ expression	2019, [110]
Lactobacillus acidophilus and Lactobacillus paracasei	Inhibit the growth of *Staphylococcus aureus*	Inhibit *Candida albicans*; prevent caries and periodontal disease	2017, [112]
Lactobavillus delbrueckii	Inhibits the growth of *Prophyromonas gingivalis*	-	2019, [113]
Lactobacillus salivarus	Efficient against *Prevotella intermedia*, *Prophyromonas gingivalis*, *Staphylococcus aureus*, and *Streptococcus salivarius*	Reduces periodontal pathogens from dental plaque, including a decrease in P. gingivalis concentration	2014, [117]
Lactobacillus bulgaricus	Inhibits *Aggregatibacter actinomycetemcomitans*	Inhibits enteropathogens	2014, [116]
Lactobacillus rhamnosus	Inhibits *Prophyromonas gingivalis* and *Fusobacterium nucleatum*	Inhibits enteropathogens	2014, [116]
Lactobacillus fermentum	Inhibits aerobe bacteria	Maintains the balance of oral microbiota	2005, [118]
Lactobacillus gasseri	Inhibits anaerobe bacteria	Stimulates natural immunity	2005, [118]
Lactobacillus johnsonii	*Aggregatibacte actinomycetemcomitans* phagocytosis	Increases macrophage activation	2018, [119]
Lactobacillus brevis	Releases arginine deiminase that inhibits the production of nitric oxide	Inhibits vascular permeability	2014, [126]
Streptococcus salivarus K12	Decrease in plaque accumulation	-	2022, [120]
Rothia	Enhances the nitrate-reduction capacity of oral communities	Prevents acidification and lactate accumulation	2020, [125]

#### 3.2.3. Antimicrobial Peptides

Antimicrobial peptides (AMPs) are a component of the innate immune system in various species. They represent molecules structured by 12 to 100 amino acids with extracellular action. There are over 1700 AMPs; some can eliminate bacteria, contributing to oral cavity homeostasis. Furthermore, they influence the host’s immune response through their immunomodulatory proprieties [127]. These AMPs can be used to develop disease markers or as therapy by targeting certain oral bacteria. The most promising ones are alfa-defensins and leucine leucine-37 (LL37). In patients with periodontal disease, cathelicidins and beta-defensins are also present in oral fluids and periodontal tissue [128]. Bacteriocins are proteins or peptides produced by bacteria. They are AMP molecules that have antimicrobial activity against other prokaryotes. There is a need to explore bacteriocin’s activity in order for it to become the next step in antibacterial treatment. This is a result of the alarming number of multi-drug-resistant bacteria. They have high temperature stability, minimal inhibitory concentrations, and low toxicity. Furthermore, they can develop bacterial resistance. The ones relevant to the oral cavity are secreted mainly by lactobacilli. Plantarcin is related to systemic health, as at the oral level it inhibits the growth of *P. gingivalis.* Some other bacteriocins, such as reuterin and nisin, have implications in periodontal disease [105]. Nisin is produced by *Lactococcus* and *Streptococcus lactis.* It interrupts cell wall formation in the targeted bacteria by generating a pore at this level. It is also more susceptible to enzymatic degradation [71].

#### 3.2.4. Nanoscale Drug Delivery Systems

Recent studies have focused on nanoscale drug delivery systems (nano-DDs) to improve bacteriocin limitations, such as immunogenicity issues or their sensitivity to proteases. Nano-DDs can change the solubility and stability of bacteriocins. They can also evade the host’s immune system and response, improving bacteriocin action against bacterial resistance mechanisms. Overall, nano-DDs can increase the bacteriocin antibacterial activity, making this association a promising new therapeutic direction [129]. Furthermore, there is a urea derivate with antimicrobial properties, especially against *Streptococcus mutans* UA159. A study conducted by Zhang and colleagues encapsulated 1,3-bis [3,5-bis(trifluoromethyl) phenylurea in poly(lactic-co-glycolic acid) (PLGA) nanoparticles. In vitro administration inhibited the growth of *S. mutans*, alongside decreasing lactic acid production, making it a promising therapeutic option for dental caries [130].

Given the accelerating bacterial antimicrobial agent resistance, searching for small molecules that elude immunological mechanisms is rapidly becoming a central research focus. 

#### 3.2.5. Oral Microbiota Transplant

Taking into consideration the success of fecal microbiota transplants in treating several gastrointestinal disorders, oral microbiome transplant exhibits great potential in oral diseases management. However, this theory has not yet been tested in humans [131]. Whereas oral microbiota transplant is not part of this natural event (involuntary transmission of oral microorganisms via saliva is a typical life occurrence), this procedure involves the relocation of healthy oral biofilms from a donor to a subject with oral disorders (caries or periodontitis) [132]. The subjects who donate are rigorous selected and should own a healthy oral environment without cariogenic bacteria presence [133]. Pozhitkov et al. [134] proposed introducing this therapeutic option to periodontitis patients. This method implies firstly performing meticulous oral treatment of the effected patients with deep cleaning, root planning, and administration of a broad-spectrum antimicrobial agent, followed by antimicrobial neutralization and local rising with a microbial suspension composed of oral microbiome harvest from a healthy donor [134]. Therefore, it is essential to conclude whether oral biofilms harvested should be transplanted directly to the affected patient or pretreated with the subsequent elimination of the pathogenic organisms prior to transplantations. Another option is the use of biofilms created in vitro which are made of naturally occurring commensal organisms [135]. Either way, this promising therapeutic method is far from being implemented, and further studies are required to determine its potential clinical value. 

#### 3.2.6. Vaccines

The advancing technology and new generations of sequencing equipment can lead to more specific systems targeting oral bacteria. Identifying new bacterial species associated with oral dysbiosis in periodontal diseases and caries could help develop vaccines. For example, a subcutaneous vaccine with formalin-killed *Porphyrominas gingivalis* promotes the production of IL-10 and transforming growth factor-beta while inhibiting T-helper 17 cells, IL-17A, and lymphocyte proliferation [136]. A specific therapeutic option against *P. gingivali*’s inflammatory immune response is parenteral or intraoral vaccination with an immunogen that targets bacterial virulence factors, such as gingipain proteinasis. The chimera (KAS2-A1) induces IgG1 antibody production and a T-helper cell type 2 response. It is possible prevention of periodontitis induced by *P. gingivalis* [136]. Unfortunately, there is no adequate human vaccine developed after the animal model. Over the years, there has been a continuous search for a vaccine targeting *Streptococcus mutants*, which is considered the leading cause of caries. Even if there are some promising options [137], studies need to be continued in order to explore new virulence targets and to start human clinical trials. Genetic engineering of different probiotics can lead to their use as vectors for oral vaccines. 

Recombinant probiotics can also target cytokines or other extracellular proteins. Furthermore, an effective vaccine for humans that targets periodontal disease prevention will need multiple gram-negative anaerobic pathogens to be selected [138]. This technology needs further studies, but it can be a helpful step in treating periodontitis and the systemic pathologies that come with it, including valvular heart disease. 

## 4. Conclusions

Microorganisms sustain all life forms. There is a close and vital relationship between the host and its pathogens. We took advantage of the emerging technology in the past years and further investigated the oral cavity microbiota. The first steps are to better understand local dysbiosis and its links to developing inflammation that leads to periodontal disease and caries. Potentially, the inflammatory process generates reactions that affect the whole body.

As we mentioned in our review paper, oral pathogens can generate systemic disease by entering the bloodstream or by triggering immunological effects at a local level. There is still a long way to go until we fully understand the mechanism through which oral microorganisms contribute to the development of non-oral pathologies, including VHD. By focusing on the prevention of periodontal disease that targets the oral microbiome, there can be a beneficial influence on the quality of life of the patients. Simple steps, such as oral hygiene and diet, can easily contribute to oral eubiosis. Furthermore, using available therapies, including prebiotics, probiotics, and antibiotic prophylaxis, opens the field in new directions. Antimicrobial peptides, nanoscale drug delivery systems, vaccines, and even oral microbiota transplant are potential new treatment options that need further study. This will generate exciting therapeutic options and brighter perspectives.

## Data Availability

No new data were created or analyzed in this study. Data sharing is not applicable to this article.

## Abbreviations

PCR	Polymerase chain reaction
VHD	Valvular heart disease
CVDs	Cardiovascular diseases
LPSs	Lipopolysaccharides
DNA	Deoxyribonucleic acid
PAMPs	Pathogen-associated molecular patterns
TLRs	Toll-like receptors
TNF-α	Tumor necrosis factor-alfa
NFk-B	Nuclear factor kappa B
IL	Interleukine
WHO	World Health Organization
RHD	Rheumatic heart disease
CAVD	Calcific aortic valve disease
RANKL	Receptor activator of nuclear factor kappa beta ligand
EGCG	Epigallocatechin-3 gallate
ECG	Epicatechin-3-gallate
AP	Antibiotic prophylaxis
IE	Infective endocarditis
AHA	American Heart Association
VSG IE	Viridans Streptococcal Group Infective Endocarditis
AMPs	Antimicrobial peptides
LL37	Leucine leucine-37
Nano-DDs	Nanoscale drug delivery systems
PLGA	1,3-bis [3,5-bis(trifluoromethyl)phenyl]urea in poly(lactic-co-glycolic acid)
